# Coexisting Iron Deficiency Anemia and Beta Thalassemia Trait: Effect of Iron Therapy on Red Cell Parameters and Hemoglobin Subtypes

**DOI:** 10.1155/2014/293216

**Published:** 2014-03-12

**Authors:** Sarika Verma, Ruchika Gupta, Madhur Kudesia, Alka Mathur, Gopal Krishan, Sompal Singh

**Affiliations:** ^1^Department of Pathology, Hindu Rao Hospital, Malka Ganj, Delhi 110007, India; ^2^Department of Pathology, All India Institute of Medical Sciences, Ansari Nagar, New Delhi 110029, India; ^3^Department of Pediatrics, Hindu Rao Hospital, Malka Ganj, Delhi 110007, India; ^4^Department of Medicine, Hindu Rao Hospital, Malka Ganj, Delhi 110007, India

## Abstract

*Background*. Coexistence of iron deficiency anemia (IDA) and beta thalassemia trait (BTT) has been the topic of few studies. However, no study from our country was found evaluating the effect of iron therapy in patients with concomitant IDA and BTT. *Methods*. Over a period of two years, 30 patients with concomitant IDA and BTT were included. All the patients had a complete blood count, serum iron studies, and thalassemia screening using BIORADTM hemoglobin testing system. The patients received oral iron therapy in appropriate dosages for a period of twenty weeks, after which all the investigations were repeated. Appropriate statistical methods were applied for comparison of pre- and posttherapy data. *Results*. All except two patients were adults with a marked female preponderance. Oral iron therapy led to statistically significant improvement in hemoglobin, red cell indices (*P* < 0.05), and marked change in serum iron, ferritin, and HbA_2_ levels (*P* < 0.001). There was a significant reduction in the total iron binding capacity levels. *Conclusion*. The present study shows the frequent occurrence of iron deficiency anemia in patients with beta thalassemia trait, which can potentially confound the diagnosis of the latter. Hence, iron deficiency should be identified and rectified in patients with suspicion of beta thalassemia trait.

## 1. Introduction

Thalassemia syndromes and iron deficiency anemia (IDA) are the two most common etiologies of microcytic hypochromic anemia in children and adults. It has long been considered that iron deficiency does not exist in thalassemia syndromes, including thalassemia major as well as trait. However, studies have shown the occurrence of iron deficiency in patients with beta thalassemia trait (BTT). Earlier authors have demonstrated lower initial hemoglobin levels in patients with coexisting IDA and BTT [1–3]. This has been explained by the lack of hemopoietic nutrients due to iron deficiency superimposing on the imbalance in globin chain synthesis [4]. Similar changes have also been shown in other red cell parameters, serum iron, ferritin, and total iron binding capacity. These changes have also been demonstrated to improve after adequate iron replacement therapy [1, 2, 5].

HbA_2_ levels have been reported to be lower in patients with coexisting IDA and BTT, with improvement in levels after iron therapy [1, 6]. However, other studies have shown no significant difference in HbA_2_ levels in such patients [7, 8]. The reduction in HbA_2_ levels in patients with concomitant BTT and IDA has been suggested to interfere in the diagnosis of the former. A recent study has hypothesized that such an occurrence can lead to these patients with BTT marrying another person with BTT with attendant risk of birth of thalassemia major child [9].

An extensive search of the available indexed English literature yielded only few Indian reports of concomitant BTT and iron deficiency [4, 10–12]. None of these studies evaluated the effect of iron therapy on red cell parameters, iron status, and hemoglobin subtypes in Indian BTT patients with concomitant iron deficiency.

The present study aimed at an extensive analysis of the effect of iron therapy on various red cell parameters and iron status in patients with concomitant IDA and BTT in our country. The study was approved by the institutional ethics committee.

## 2. Materials and Methods

This prospective study included patients attending the hematology clinics of departments of pediatrics and medicine at a tertiary care centre over a period of two years (Dec 2006–Nov 2008). Patients with HbA_2_ levels >3.7% with low serum ferritin (<10 ng/mL for females and <16 ng/mL for males), normal random blood sugar levels, and no evidence of other hemoglobinopathy were included in the study. Cases with lower HbA_2_ levels, other hemoglobinopathies, abnormal blood sugar levels, and normal or high ferritin levels were excluded from the study. Written informed consent was taken from the patients/guardian (in case of pediatric patients).

All the included patients underwent complete blood counts using an automated hematology analyzer, thalassemia screening by high performance liquid chromatography (HPLC) using BIORAD Variant hemoglobin testing system (BIORAD Laboratories, Hercules, USA), and serum iron studies, including serum iron and total iron binding capacity, TIBC using a kit (POINTE SCIENTIFIC Inc., USA), and serum ferritin by a microtiter ELISA kit (DiaMed EuroGen, Belgium).

After the initial investigations, these patients were initiated on oral iron therapy (tab ferrous sulphate: children 1.5–2 mg/kg body weight elemental iron tds; adults 60 mg/day elemental iron tds) for twenty weeks. On completion of therapy, all the investigations were repeated.

The pre- and posttherapy data was analyzed using appropriate statistical tests. The change in hematological parameters, serum iron, TIBC, and hemoglobin subtypes were analyzed using paired “*t*” test. Serum ferritin values were compared using the nonparametric Mann-Whitney test. A *P* value of <0.05 was considered significant.

## 3. Results

Over the study period, 30 patients of BTT with concomitant iron deficiency were included. Majority of our patients (28, 93.3%) were adults with a mean age of 29.2 ± 9.03 years. Only two patients were children, 10 years and 13 years of age. There was a marked female preponderance (5 males, 25 females, M : F 1 : 5).

### 3.1. Pretherapy Parameters

The mean hemoglobin level was 9.8 ± 1.1 g/dL with a range of 8.2–11.2 g/dL. Serum iron ranged between 20.0 and 58.0 *μ*g/dL (mean 47.1 ± 8.17 *μ*g/dL) while mean TIBC was 502 *μ*g/dL (±34.17 *μ*g/dL). Serum ferritin levels ranged between 1.1 and 9.6 ng/mL (median 6.75 ng/mL). These parameters are tabulated in Table 1.

HbF values ranged from 0.0 to 3.7% (mean 0.9 ± 0.8%) while HbA_2_ levels varied between 3.8 and 7% (mean 5.4 ± 0.86%). HbA1c before iron therapy ranged from 4.7 to 6.22% (mean 5.4 ± 0.45%).

### 3.2. Posttherapy Findings and Statistical Analysis

The mean hemoglobin level rose to 10.8 ± 1.11 g/dL and this difference was statistically significant (*P* < 0.001). Similarly, serum iron levels rose to a mean of 70.3 ± 8.15 *μ*g/dL with statistically significant difference (*P* < 0.001). Serum ferritin also showed a significant increase after therapy, while TIBC reduced.

**Figure 1:**
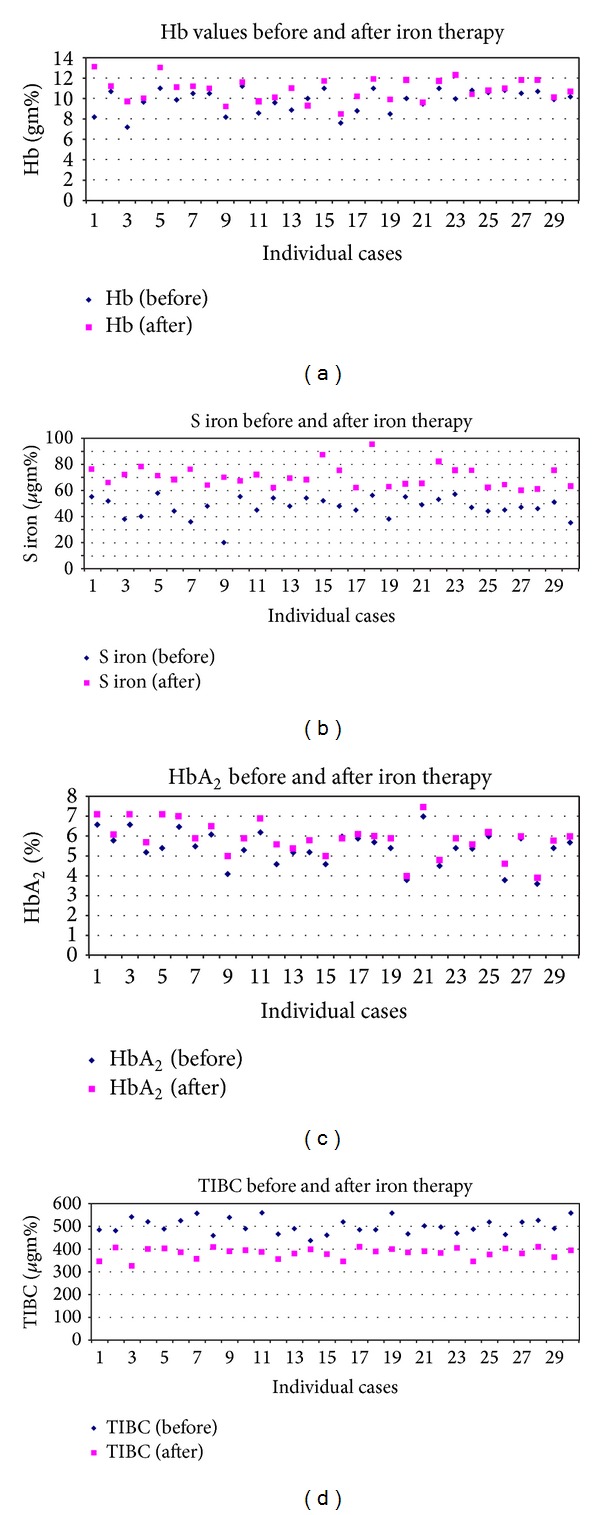
Graphs demonstrating the change in hemoglobin values (a), serum iron (b), HbA_2_ levels (c), and TIBC values after iron therapy.

**Figure 2:**
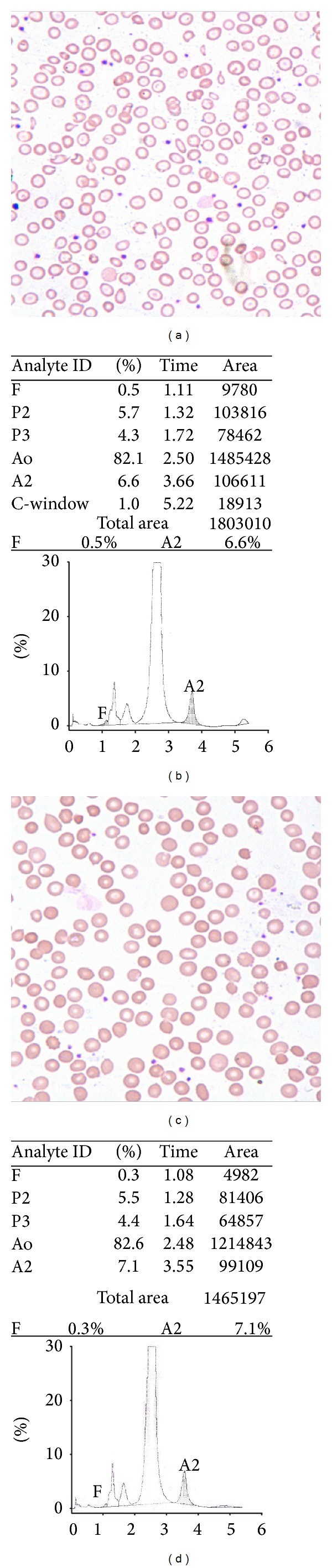
Peripheral smear from a patient with concomitant iron deficiency and beta thalassemia trait before iron therapy (a, Giemsa ×400). HPLC graph of the same patient before iron therapy (b). Peripheral smear of the same case as in an after iron therapy (b, Giemsa ×400). HPLC graph of the same patient after iron therapy (d).

HbF levels remained largely unchanged after iron therapy while HbA_2_ values showed significant rise after therapy (*P* = 0.04). These results are summarised in Table 1 and depicted in Figure 1. The peripheral smears and HPLC graphs of an illustrative case with concomitant IDA and BTT are shown in Figure 2.

## 4. Discussion

Iron deficiency anemia and thalassemia syndromes, especially beta thalassemia trait (BTT), are the two most frequent microcytic hypochromic anemias highly prevalent in countries like India [13, 14]. The National Family Health Survey (NFHS-3) of 2011 reveals the prevalence of anemia as 70–80% in children, 70% in pregnant women, and 24% in adult men. The prevalence of BTT has been cited as 3.5–10% in India [15, 16].

Iron status in BTT has been an area of interest for many authors. Due to the frequent iron overload in thalassemia major patients, it was earlier believed that iron deficiency does not exist in BTT also. However, a study by Alperin et al. in 1976 included 33 patients of BTT with evidence of iron deficiency and showed lower initial hemoglobin levels, which improved after iron replacement therapy [1]. Similar findings were reported by other authors as well [2, 17]. A recent study from south India also showed lower hemoglobin levels in BTT with iron deficiency than those without [3]. In our study also, the mean hemoglobin value (9.8 ± 1.1 g/dL) was lower than expected in uncomplicated BTT. Saraya et al., in their study, attempted to explain that iron deficiency in BTT leads to lack of hemopoietic nutrients in addition to imbalance in globin chain synthesis resulting in further reduction in hemoglobin production [4]. Harthoorn-Lasthuizen et al. concluded that hemoglobin values in BTT can neither indicate the presence of concomitant iron deficiency nor reflect the severity of iron deficiency [18]. Hemoglobin levels of our patients improved significantly (*P* < 0.001) after iron replacement therapy. These results are similar to those reported in previous studies [1, 5, 17]. Similarly, red cell indices, namely, MCV, MCH, MCHC, and RDW-CV, also showed significant improvement after iron therapy. Red cell counts, in our study, were in the near-normal range (4.9 ± 0.85 million/mm^3^) prior to iron therapy. BTT is associated with relative erythrocytosis, while iron deficiency leads to low RBC counts. Coexistence of these two conditions led to RBC counts being near the normal range in our study. The RBC counts increased after iron therapy, although the change was not significant. One reason may be that the initial RBC count was higher than expected in uncomplicated iron deficiency anemia, as described in a study by El-Agouza et al. [5].

The parameters of iron status, as expected, improved after sufficient iron replacement therapy. In our patients, serum iron increased significantly with values reaching the normal range. TIBC, which was high prior to iron therapy, normalized indicating good compliance to iron therapy. We used serum ferritin levels <15 ng/mL as a cutoff for iron deficiency, as in previous studies [2–4]. Saraya et al. reported that low serum ferritin was more frequently seen in female patients with BTT than male patients and recommended iron supplementation for females [4]. Serum ferritin levels improved significantly and reached normal values after iron therapy. These results are similar to previous studies [5].

Among hemoglobin subtypes, HbF values did not show any significant change after iron therapy. This is similar to previous studies by El-Agouza et al. and Wasi et al. [5, 6]. There have been conflicting reports of the effect of iron deficiency on HbA_2_ levels. Few authors have reported a significantly lower HbA_2_ levels in patients with concomitant BTT and iron deficiency compared to those with uncomplicated BTT [1, 5, 6], while others have failed to elicit such a difference [7, 8]. One of the reasons of these discrepant results appears to be the variance in cutoff level of serum ferritin to define iron deficiency. Passarello et al. considered 30 *μ*g/L as cutoff for iron deficiency [8], which in our opinion is not suitable since this level defines negative iron balance and not iron deficiency [19]. Several hypotheses have been put forward for the diminished HbA_2_ levels in iron deficiency. Wasi et al. have suggested that beta chains may be more competitive than delta chains in heme binding leading to less HbA_2_ formation in heme deficiency. Lack of iron may also interfere with delta chain synthesis [6]. Other authors have suggested that low HbA_2_ levels could be due to decreased transcription or translation of delta gene [5, 20]. Harthoorn-Lasthuizen et al. hypothesized that lack of iron reduces the synthesis of alpha globin chains compared to nonalpha chains. With limited supply of beta chains in BTT, beta chains compete more effectively for alpha chains than delta chains [10, 18].

HbA_2_ levels, in our patients, improved significantly after iron replacement therapy. Similar results have been reported by earlier authors [5, 6, 10]. These findings underscore the importance of treating iron deficiency for a minimum of 16 weeks, especially if HbA_2_ levels are borderline.

An extensive search of the available indexed English literature yielded only few Indian reports of concomitant BTT and iron deficiency [4, 10–12]. Most of these studies only evaluated the red cell parameters in such patients. However, no study was found evaluating the effect of iron therapy on red cell parameters, iron status, and hemoglobin subtypes in Indian BTT patients with concomitant iron deficiency. The present study is the first such extensive analysis from Indian subcontinent, to the best of our knowledge. Although we included only patients of BTT (HbA_2_ > 3.7%) with concomitant iron deficiency, these results can be extrapolated to patients with HbA_2_ levels <3.7% due to effect of iron deficiency and expect similar changes in various parameters after iron replacement therapy.

A recent study from Pakistan reported low HbA_2_ levels in BTT with concomitant iron deficiency leading to diagnostic difficulties. The authors suggested that such patients, who may be diagnosed as normal on hemoglobin electrophoresis or HPLC, could marry a person with BTT and lead to birth of children with beta thalassemia major. Such an occurrence poses a serious hindrance for the thalassemia prevention program. Hence, the authors concluded that coexisting pathological conditions should be identified before reporting the hemoglobin electrophoresis or HPLC as normal, especially in countries with high incidence of both iron deficiency and BTT [9].

In conclusion, the present study highlights the coexistence of iron deficiency anemia and beta thalassemia trait in Indian patients. The diagnosis of beta thalassemia trait in such patients may be confounded by reduction in HbA_2_ levels. Hence, iron deficiency should be identified and corrected in patients with high suspicion of beta thalassemia trait, especially if HbA_2_ levels are within normal range.

## Figures and Tables

**Table 1:** Pre- and posttherapy hematological and biochemical parameters.

Parameter	Pretherapy	Posttherapy	*P* value
Hemoglobin (g/dL)	9.8 ± 1.1	10.8 ± 1.11	**<0.001**
RBC count (million/mm^3^)	4.9 ± 0.85	5.1 ± 0.76	0.35
Mean corpuscular volume, MCV (fL)	64.0 ± 6.44	67.9 ± 4.84	**0.01**
Mean corpuscular hemoglobin, MCH (pg)	20.0 ± 3.0	21.5 ± 3.01	**0.04**
Mean corpuscular hemoglobin concentration	30.2 ± 2.35	31.4 ± 2.11	**0.04**
Red cell distribution width, RDW-CV (%)	18.6 ± 2.13	16.08 ± 1.53	**<0.001**
Serum iron (*μ*g/dL)	47.1 ± 8.17	70.3 ± 8.15	**<0.001**
TIBC (*μ*g/dL)	502 ± 34.17	383.2 ± 22.18	**<0.001**
Ferritin (ng/mL)*	6.75	25.9	**<0.001**
	(QD 1.175)	(QD 10.96)	
HbF (%)	0.9 ± 0.8	0.9 ± 0.84	0.98
HbA_2_ (%)	5.4 ± 0.86	5.8 ± 0.87	**0.04**

*Ferritin in median values; QD: quartile deviation.
